# Novel gene mutations underlying two new cases of ALPS 0 syndrome

**Published:** 2011-01-10

**Authors:** MR Barbouche, I Ben Mustapha, N Dhouib, N Mattoussi, R Riahi, B Larguèche, S Ben Becheur, M Béjaoui

**Affiliations:** 1 Department of Immunology, Institut Pasteur de Tunis, Tunis, Tunisia; 2 Bone Marrow Transplantation Center, Tunis, Tunisia; 3 Department of Pediatrics, Children Hospital, Tunis, Tunisia

## 

Autoimmune lymphoproliferative syndrome (ALPS) is classified among primary immune deficiencies. This prototypic disorder of impaired apoptosis in humans is characterized mainly by autoimmune features and lymphoproliferation. ALPS type 0, caused by homozygous null mutations of the CD95 gene (Fas) leading to a severe ALPS phenotype, is a very rare subgroup with only 3 published cases. Here, we describe two North African male infants (a Tunisian and a Libyan) with ALPS 0 syndrome. They are born to consanguineous parents and were both investigated at age 10 months for lymphadenopathy, splenomegaly and auto-immune hemolytic anemia. The two patients showed increased levels of serum immunoglobulins. Immunophenotyping showed a high percentage (15% and 11% respectively) of CD4-CD8-TCRαβ+ and a complete absence of Fas expression. As previously described, we found elevated levels of IL-10 protein (1934pg/ml and 771pg/ml respectively) and sFasLigand (1.7ng/ml and >5ng/ml respectively) in their plasma. RT-PCR analysis demonstrated the skipping of exon 6, coding for the Fas transmembrane domain, for both patients. Sequencing analysis of the Fas gene showed that one patient has a homozygous substitution 16 nucleotides upstream of the 3’ acceptor splice site of intron 5 (c.506-16A>G). This mutation may result in profound defects of gene expression at the level of pre-mRNA splicing as it breaks a potential branch point sequence. The other patient has a homozygous substitution within exon 6 (c.514C>T) which alters a potential exonic regulatory splicing site. These predictions have been obtained using human splicing finder software. The parents DNA was available for one patient and both were healthy heterozygous. Furthermore, these mutations were not found in 30 healthy individuals excluding a potential polymorphism. The two identified, previously non described mutations result in absent surface expression of the Fas receptor, precluding binding of FasL and leading to severe clinical symptoms. They expand on the few previously reported ALPS 0 cases and provide further insights into Fas gene molecular defects.

